# Poly[aqua­[μ_5_-5-(isonicotinamido)­isophthalato][μ_4_-5-(isonicotinamido)­isophthalato]cerium(III)silver(I)]

**DOI:** 10.1107/S1600536811027668

**Published:** 2011-07-23

**Authors:** Xue Nie, Jing-Nian Qu

**Affiliations:** a Department of Chemistry and Materials Science, Hengyang, Hunan 421008, People’s Republic of China

## Abstract

The 4*d*–4*f* heteronuclear title complex, [AgCe(C_14_H_8_N_2_O_5_)_2_(H_2_O)]_*n*_, has a three-dimensional framework structure, generated by the carboxyl­ate and pyridyl groups of the 5-(isonicotinamido)­isophthalate (INAIP) ligands bridging the metal ions. The Ce^III^ atom is coordinated by eight O atoms from six INAIP ligands and a water mol­ecule in a distorted tricapped trigonal–prismatic geometry, while the Ag^I^ atom has a distorted trigonal–planar AgN_2_O geometry. O—H⋯O and N—H⋯O hydrogen bonds and π–π inter­actions between the pyridine and benzene rings [centroid–centroid distances = 3.642 (4) and 3.624 (3) Å] stabilize the crystal structure.

## Related literature

For background to coordination polymers, see: Abourahma *et al.* (2002 ▶); Costes *et al.* (2004 ▶); Kapoor *et al.* (2002 ▶). For background to lanthanide and transition metal heterometallic compounds, see: Chen *et al.* (2010 ▶); Cheng *et al.* (2006 ▶); Lin *et al.* (2009 ▶); Zhang *et al.* (2005 ▶).
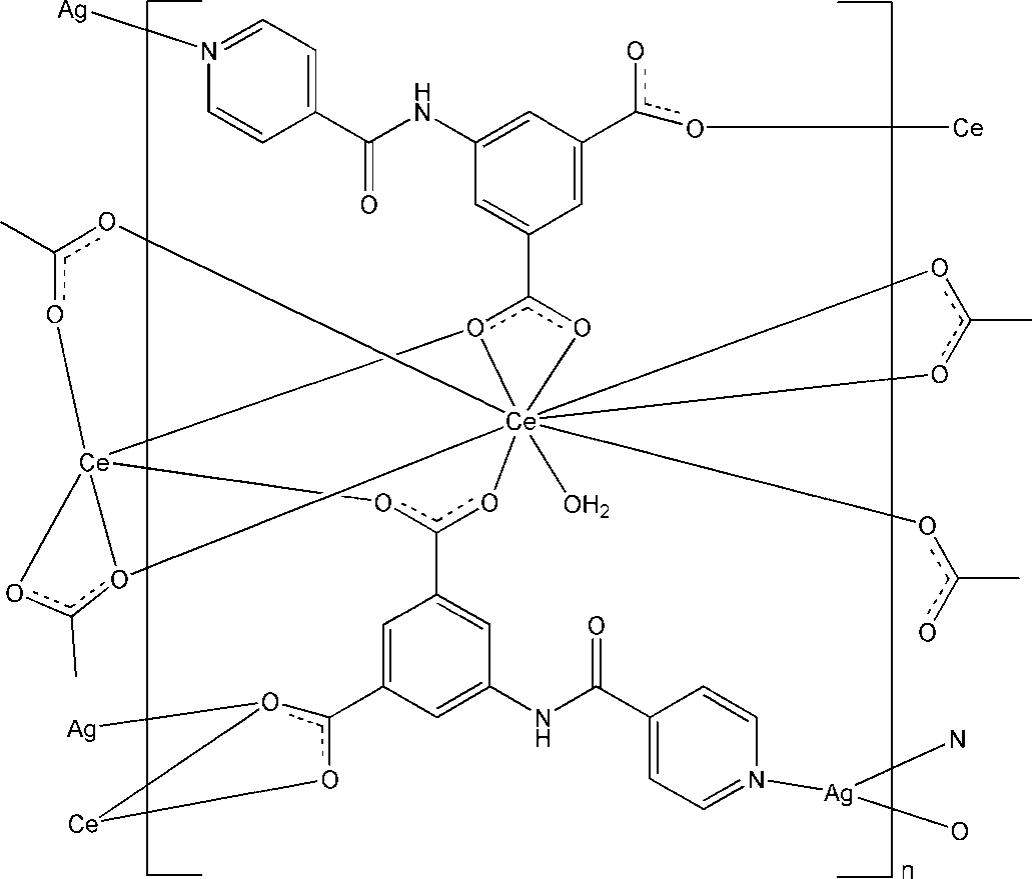


## Experimental

### 

#### Crystal data

[AgCe(C_14_H_8_N_2_O_5_)_2_(H_2_O)]
*M* *_r_* = 834.45
Triclinic, 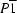

*a* = 10.4869 (14) Å
*b* = 11.1540 (15) Å
*c* = 13.7276 (18) Å
α = 107.853 (1)°
β = 106.778 (2)°
γ = 102.885 (2)°
*V* = 1375.2 (3) Å^3^
*Z* = 2
Mo *K*α radiation
μ = 2.42 mm^−1^
*T* = 293 K
0.20 × 0.14 × 0.10 mm

#### Data collection

Bruker APEX CCD diffractometer
Absorption correction: multi-scan (*SADABS*; Bruker, 2001 ▶) *T*_min_ = 0.643, *T*_max_ = 0.794
6903 measured reflections
4777 independent reflections
4178 reflections with *I* > 2σ(*I*)
*R*_int_ = 0.053

#### Refinement

*R*[*F* ^2^ > 2σ(*F* ^2^)] = 0.035
*wR*(*F* ^2^) = 0.081
*S* = 0.97
4777 reflections
406 parameters
H-atom parameters constrained
Δρ_max_ = 1.40 e Å^−3^
Δρ_min_ = −1.44 e Å^−3^

### 

Data collection: *SMART* (Bruker, 2007 ▶); cell refinement: *SAINT* (Bruker, 2007 ▶); data reduction: *SAINT*; program(s) used to solve structure: *SHELXTL* (Sheldrick, 2008 ▶); program(s) used to refine structure: *SHELXTL*; molecular graphics: *SHELXTL*; software used to prepare material for publication: *SHELXTL*.

## Supplementary Material

Crystal structure: contains datablock(s) global, I. DOI: 10.1107/S1600536811027668/hy2446sup1.cif

Structure factors: contains datablock(s) I. DOI: 10.1107/S1600536811027668/hy2446Isup2.hkl

Additional supplementary materials:  crystallographic information; 3D view; checkCIF report

## Figures and Tables

**Table 1:** Hydrogen-bond geometry (Å, °)

*D*—H⋯*A*	*D*—H	H⋯*A*	*D*⋯*A*	*D*—H⋯*A*
O1*W*—H1*WA*⋯O5^i^	0.85	2.08	2.836 (5)	147
O1*W*—H1*WB*⋯O4^ii^	0.85	2.01	2.691 (6)	137
N2—H2⋯O10^iii^	0.86	2.01	2.788 (7)	149
N4—H4⋯O4^i^	0.86	2.08	2.926 (6)	167

Symmetry codes: (i) 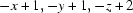; (ii) 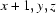; (iii) 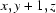.

**Fig. 1.**
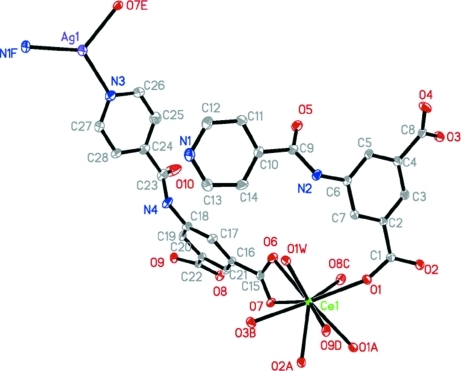
The asymmetric unit of the title compound. Displacement ellipsoids are drawn at the 30% probability level. [Symmetry codes: (A) 1-x, 1-y, 1-z; (B) 1+x, y, z; (C) 1-x, -y, 1-z; (D) x, 1+y, z; (E) x, y, 1+z; (F) 2-x, 1-y, 3-z.]

**Fig. 2.**
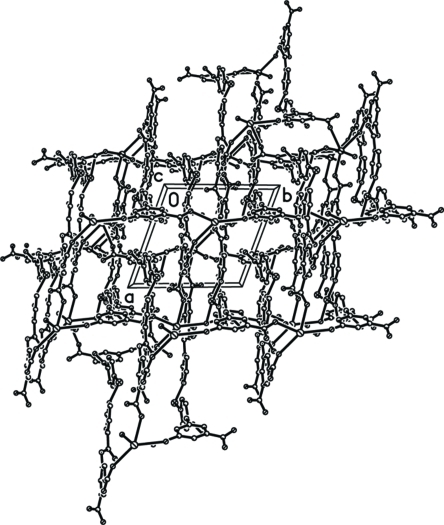
A view of the three-dimensional framework of the title compound.

## supplementary crystallographic information

### Comment

In recent years, coordination polymeric frameworks have attracted great attention because of their potential applications and intriguing structure topologies (Abourahma *et al.*, 2002; Costes *et al.*, 2004; Kapoor *et al.*, 2002). To obtain d-f coordination polymers is more important. In general, multidentate ligands containing both N- and O-donor atoms are usually employed in the construction of lanthanide (Ln) and transition metal (M) heterometallic structures, in keeping with the typical coordination behaviors of Ln and M ions under different reaction conditions (Cheng *et al.*, 2006; Lin *et al.*, 2009; Zhang *et al.*, 2005). Compared with other N-heterocyclic acids, 5-(isonicotinamido)isophthalic acid (H_2_INAIP) shows richer coordination modes due to its two carboxylate groups and one pyridyl group, and accordingly it is an excellent candidate for the construction of metal-organic frameworks (Chen *et al.*, 2010). In this paper, we report the synthesis and structure of the title complex (Fig. 1), a 4d-4f heterometallic coordination polymer.

It is interesting that two INAIP ligands exhibit different coordination modes: one coordinated to three Ce^III^ atoms and two Ag^I^ atoms while the other coordinated to three Ce^III^ atoms and one Ag^I^ atom, originated from the different coordination modes of the carboxylate groups. If the Ag—N and Ag—O connections are neglected, a two-dimensional (4, 4) bilayer network is formed by the Ce^III^–carboxylate coordination. The (4, 4) nets are linked together by Ag—N and Ag—O coordination interactions, forming a complicated three-dimensional coordination net (Fig. 2).

### Experimental

A mixture of Ce(NO_3_)_3_.6H_2_O (22.0 mg, 0.05 mmol), H_2_INAIP (28.6 mg, 0.1 mmol), AgNO_3_ (8.5 mg, 0.05 mmol), NaOH (6.0 mg, 0.15 mmol) and H_2_O (10 ml) was heated in a 16 ml Teflon-lined reaction vessel at 453 K for 4 d. The reaction mixture was cooled to room temperature over a period of 40 h. The product was collected by filtration, washed with H_2_O and air dried.

### Refinement

H atoms bonded to C and N atoms were positioned geometrically and refined as riding atoms, with C—H = 0.93 and N—H = 0.86 Å and *U*_iso_(H) = 1.2*U*_eq_(C, N). The water H atoms were found from a difference Fourier maps and refined as riding, with O—H = 0.85 Å and with *U*_iso_(H) = 1.2*U*_eq_(O). The highest residual electron density was found at 0.93 Å from Ag1 atom and the deepest hole at 0.86 Å from Ag1 atom.

### Crystal data

**Table:**

[AgCe(C_14_H_8_N_2_O_5_)_2_(H_2_O)]	*Z* = 2
*M**_r_* = 834.45	*F*(000) = 814
Triclinic, *P*1	*D*_x_ = 2.015 Mg m^−^^3^
Hall symbol: -P 1	Mo *K*α radiation, λ = 0.71073 Å
*a* = 10.4869 (14) Å	Cell parameters from 4777 reflections
*b* = 11.1540 (15) Å	θ = 2.0–25.0°
*c* = 13.7276 (18) Å	µ = 2.42 mm^−^^1^
α = 107.853 (1)°	*T* = 293 K
β = 106.778 (2)°	Block, colorless
γ = 102.885 (2)°	0.20 × 0.14 × 0.10 mm
*V* = 1375.2 (3) Å^3^	

### Data collection

**Table:**

Bruker APEX CCD diffractometer	4777 independent reflections
Radiation source: fine-focus sealed tube	4178 reflections with *I* > 2σ(*I*)
graphite	*R*_int_ = 0.053
φ and ω scans	θ_max_ = 25.0°, θ_min_ = 2.0°
Absorption correction: multi-scan (*SADABS*; Bruker, 2001)	*h* = −12→12
*T*_min_ = 0.643, *T*_max_ = 0.794	*k* = −11→13
6903 measured reflections	*l* = −16→12

### Refinement

**Table:**

Refinement on *F*^2^	Primary atom site location: structure-invariant direct methods
Least-squares matrix: full	Secondary atom site location: difference Fourier map
*R*[*F*^2^ > 2σ(*F*^2^)] = 0.035	Hydrogen site location: inferred from neighbouring sites
*wR*(*F*^2^) = 0.081	H-atom parameters constrained
*S* = 0.97	*w* = 1/[σ^2^(*F*_o_^2^) + (0.0347*P*)^2^] where *P* = (*F*_o_^2^ + 2*F*_c_^2^)/3
4777 reflections	(Δ/σ)_max_ = 0.001
406 parameters	Δρ_max_ = 1.40 e Å^−^^3^
0 restraints	Δρ_min_ = −1.44 e Å^−^^3^

### Fractional atomic coordinates and isotropic or equivalent isotropic displacement parameters (Å^2^)

**Table:**

	*x*	*y*	*z*	*U*_iso_*/*U*_eq_	
Ag1	0.88225 (5)	0.23184 (7)	1.55049 (4)	0.06266 (18)	
Ce1	0.63603 (3)	0.45288 (2)	0.61285 (2)	0.01607 (9)	
C1	0.3260 (5)	0.5683 (4)	0.5961 (4)	0.0200 (10)	
C2	0.2981 (5)	0.5843 (4)	0.7000 (4)	0.0202 (10)	
C3	0.1600 (5)	0.5553 (4)	0.6961 (4)	0.0222 (10)	
H3	0.0837	0.5240	0.6288	0.027*	
C4	0.1376 (5)	0.5735 (5)	0.7933 (4)	0.0234 (10)	
C5	0.2530 (5)	0.6195 (5)	0.8932 (4)	0.0247 (11)	
H5	0.2378	0.6312	0.9584	0.030*	
C6	0.3879 (5)	0.6476 (4)	0.8964 (4)	0.0209 (10)	
C7	0.4116 (5)	0.6312 (4)	0.8000 (4)	0.0223 (10)	
H7	0.5037	0.6517	0.8023	0.027*	
C8	−0.0077 (5)	0.5580 (5)	0.7942 (4)	0.0240 (11)	
C9	0.5223 (5)	0.6542 (5)	1.0769 (4)	0.0236 (11)	
C10	0.6688 (5)	0.7096 (5)	1.1656 (4)	0.0260 (11)	
C11	0.6840 (6)	0.7319 (6)	1.2735 (4)	0.0398 (14)	
H11	0.6052	0.7199	1.2925	0.048*	
C12	0.8176 (6)	0.7723 (7)	1.3521 (4)	0.0469 (16)	
H12	0.8272	0.7904	1.4252	0.056*	
C13	0.9185 (6)	0.7691 (7)	1.2276 (5)	0.0481 (16)	
H13	0.9993	0.7828	1.2112	0.058*	
C14	0.7895 (6)	0.7313 (6)	1.1433 (4)	0.0418 (14)	
H14	0.7840	0.7205	1.0721	0.050*	
C15	0.6561 (5)	0.2158 (5)	0.6570 (4)	0.0240 (11)	
C16	0.6919 (5)	0.1164 (4)	0.7036 (4)	0.0217 (10)	
C17	0.7361 (5)	0.1512 (5)	0.8175 (4)	0.0258 (11)	
H17	0.7431	0.2353	0.8638	0.031*	
C18	0.7693 (5)	0.0620 (5)	0.8619 (4)	0.0252 (11)	
C19	0.7550 (5)	−0.0648 (5)	0.7921 (4)	0.0241 (11)	
H19	0.7773	−0.1255	0.8219	0.029*	
C20	0.7076 (5)	−0.1014 (4)	0.6784 (4)	0.0215 (10)	
C21	0.6768 (5)	−0.0101 (4)	0.6344 (4)	0.0223 (10)	
H21	0.6460	−0.0340	0.5582	0.027*	
C22	0.6810 (5)	−0.2419 (5)	0.6031 (4)	0.0212 (10)	
C23	0.7450 (6)	0.0246 (5)	1.0210 (4)	0.0356 (13)	
C24	0.7834 (6)	0.0781 (5)	1.1428 (4)	0.0318 (12)	
C25	0.6756 (6)	0.0579 (6)	1.1812 (5)	0.0428 (14)	
H25	0.5819	0.0151	1.1312	0.051*	
C26	0.7064 (7)	0.1008 (6)	1.2925 (5)	0.0437 (14)	
H26	0.6321	0.0865	1.3165	0.052*	
C27	0.9447 (6)	0.1839 (6)	1.3330 (5)	0.0452 (15)	
H27	1.0375	0.2270	1.3846	0.054*	
C28	0.9196 (6)	0.1435 (6)	1.2209 (4)	0.0384 (14)	
H28	0.9950	0.1608	1.1985	0.046*	
N1	0.9338 (5)	0.7871 (5)	1.3303 (4)	0.0409 (12)	
N2	0.5085 (4)	0.6990 (4)	0.9965 (3)	0.0249 (9)	
H2	0.5787	0.7647	1.0061	0.030*	
N3	0.8395 (5)	0.1627 (5)	1.3687 (4)	0.0401 (12)	
N4	0.8112 (5)	0.0994 (4)	0.9783 (3)	0.0285 (10)	
H4	0.8810	0.1721	1.0226	0.034*	
O1	0.4458 (3)	0.5621 (3)	0.5960 (2)	0.0233 (7)	
O2	0.2324 (4)	0.5616 (4)	0.5135 (3)	0.0329 (9)	
O3	−0.1121 (3)	0.4777 (3)	0.7074 (3)	0.0280 (8)	
O4	−0.0163 (4)	0.6309 (4)	0.8809 (3)	0.0341 (9)	
O5	0.4260 (4)	0.5710 (3)	1.0791 (3)	0.0316 (8)	
O6	0.6166 (4)	0.3009 (3)	0.7118 (3)	0.0280 (8)	
O7	0.6661 (4)	0.2109 (3)	0.5664 (2)	0.0241 (7)	
O8	0.6024 (4)	−0.2802 (3)	0.5023 (3)	0.0270 (8)	
O9	0.7369 (3)	−0.3125 (3)	0.6448 (3)	0.0259 (8)	
O10	0.6548 (5)	−0.0862 (4)	0.9625 (3)	0.0639 (14)	
O1W	0.7032 (3)	0.5815 (3)	0.8217 (3)	0.0266 (8)	
H1WB	0.7778	0.5704	0.8568	0.032*	
H1WA	0.6372	0.5446	0.8383	0.032*	

### Atomic displacement parameters (Å^2^)

**Table:**

	*U*^11^	*U*^22^	*U*^33^	*U*^12^	*U*^13^	*U*^23^
Ag1	0.0349 (3)	0.1311 (5)	0.0321 (3)	0.0401 (3)	0.0142 (2)	0.0360 (3)
Ce1	0.01710 (15)	0.01732 (14)	0.01439 (14)	0.00767 (11)	0.00504 (11)	0.00675 (10)
C1	0.022 (3)	0.018 (2)	0.019 (2)	0.006 (2)	0.008 (2)	0.005 (2)
C2	0.020 (3)	0.025 (2)	0.020 (2)	0.012 (2)	0.009 (2)	0.011 (2)
C3	0.020 (3)	0.025 (2)	0.017 (2)	0.008 (2)	0.003 (2)	0.007 (2)
C4	0.022 (3)	0.027 (3)	0.021 (3)	0.009 (2)	0.008 (2)	0.009 (2)
C5	0.026 (3)	0.033 (3)	0.015 (2)	0.015 (2)	0.006 (2)	0.009 (2)
C6	0.021 (3)	0.021 (2)	0.018 (2)	0.007 (2)	0.004 (2)	0.008 (2)
C7	0.019 (3)	0.027 (3)	0.024 (3)	0.010 (2)	0.009 (2)	0.012 (2)
C8	0.022 (3)	0.030 (3)	0.020 (3)	0.009 (2)	0.006 (2)	0.013 (2)
C9	0.024 (3)	0.026 (3)	0.015 (2)	0.006 (2)	0.005 (2)	0.005 (2)
C10	0.028 (3)	0.027 (3)	0.025 (3)	0.011 (2)	0.008 (2)	0.013 (2)
C11	0.026 (3)	0.072 (4)	0.025 (3)	0.019 (3)	0.011 (2)	0.021 (3)
C12	0.035 (4)	0.081 (5)	0.023 (3)	0.024 (3)	0.006 (3)	0.020 (3)
C13	0.025 (3)	0.082 (5)	0.040 (4)	0.017 (3)	0.011 (3)	0.030 (3)
C14	0.032 (3)	0.070 (4)	0.022 (3)	0.011 (3)	0.011 (3)	0.021 (3)
C15	0.021 (3)	0.024 (3)	0.029 (3)	0.010 (2)	0.008 (2)	0.015 (2)
C16	0.022 (3)	0.019 (2)	0.026 (3)	0.008 (2)	0.008 (2)	0.012 (2)
C17	0.034 (3)	0.020 (2)	0.024 (3)	0.010 (2)	0.012 (2)	0.008 (2)
C18	0.029 (3)	0.024 (3)	0.019 (2)	0.006 (2)	0.009 (2)	0.008 (2)
C19	0.027 (3)	0.023 (2)	0.024 (3)	0.011 (2)	0.005 (2)	0.013 (2)
C20	0.023 (3)	0.018 (2)	0.024 (3)	0.007 (2)	0.008 (2)	0.010 (2)
C21	0.023 (3)	0.023 (2)	0.020 (2)	0.008 (2)	0.006 (2)	0.010 (2)
C22	0.017 (3)	0.026 (2)	0.022 (3)	0.006 (2)	0.009 (2)	0.011 (2)
C23	0.041 (3)	0.029 (3)	0.030 (3)	0.007 (3)	0.009 (3)	0.011 (3)
C24	0.040 (3)	0.030 (3)	0.028 (3)	0.011 (3)	0.013 (3)	0.015 (2)
C25	0.034 (3)	0.048 (3)	0.032 (3)	0.006 (3)	0.005 (3)	0.011 (3)
C26	0.041 (4)	0.063 (4)	0.035 (3)	0.020 (3)	0.020 (3)	0.024 (3)
C27	0.035 (4)	0.076 (4)	0.033 (3)	0.026 (3)	0.013 (3)	0.028 (3)
C28	0.041 (4)	0.058 (4)	0.028 (3)	0.023 (3)	0.018 (3)	0.024 (3)
N1	0.026 (3)	0.064 (3)	0.028 (3)	0.016 (2)	0.004 (2)	0.018 (2)
N2	0.019 (2)	0.030 (2)	0.020 (2)	0.0024 (18)	0.0017 (18)	0.0124 (18)
N3	0.043 (3)	0.061 (3)	0.030 (3)	0.029 (3)	0.018 (2)	0.023 (2)
N4	0.036 (3)	0.023 (2)	0.018 (2)	0.0039 (19)	0.0031 (19)	0.0088 (18)
O1	0.0193 (18)	0.0286 (18)	0.0219 (17)	0.0091 (15)	0.0082 (15)	0.0089 (15)
O2	0.028 (2)	0.060 (2)	0.0197 (18)	0.0253 (19)	0.0103 (16)	0.0198 (18)
O3	0.0173 (18)	0.038 (2)	0.0197 (18)	0.0067 (16)	0.0035 (15)	0.0058 (16)
O4	0.0186 (19)	0.049 (2)	0.0256 (19)	0.0095 (17)	0.0089 (16)	0.0045 (17)
O5	0.025 (2)	0.042 (2)	0.0259 (19)	0.0067 (17)	0.0073 (16)	0.0177 (17)
O6	0.043 (2)	0.0247 (18)	0.0283 (19)	0.0201 (17)	0.0185 (17)	0.0148 (16)
O7	0.032 (2)	0.0250 (17)	0.0207 (18)	0.0128 (15)	0.0115 (15)	0.0124 (15)
O8	0.031 (2)	0.0209 (17)	0.0209 (18)	0.0057 (15)	0.0036 (16)	0.0054 (15)
O9	0.0257 (19)	0.0216 (17)	0.0313 (19)	0.0120 (15)	0.0069 (16)	0.0128 (15)
O10	0.083 (4)	0.044 (2)	0.033 (2)	−0.021 (2)	0.020 (2)	0.007 (2)
O1W	0.0233 (19)	0.0369 (19)	0.0245 (18)	0.0140 (16)	0.0117 (15)	0.0134 (16)

### Geometric parameters (Å, °)

**Table:**

Ag1—N1^i^	2.239 (4)	C13—N1	1.318 (7)
Ag1—N3	2.246 (4)	C13—C14	1.374 (8)
Ag1—O7^ii^	2.306 (3)	C13—H13	0.9300
Ce1—O9^iii^	2.439 (3)	C14—H14	0.9300
Ce1—O8^iv^	2.483 (3)	C15—O6	1.247 (5)
Ce1—O6	2.485 (3)	C15—O7	1.264 (5)
Ce1—O3^v^	2.493 (3)	C15—C16	1.503 (6)
Ce1—O2^vi^	2.499 (3)	C16—C21	1.383 (6)
Ce1—O1	2.551 (3)	C16—C17	1.393 (6)
Ce1—O1W	2.576 (3)	C17—C18	1.374 (6)
Ce1—O1^vi^	2.685 (3)	C17—H17	0.9300
Ce1—O7	2.690 (3)	C18—C19	1.390 (6)
C1—O2	1.237 (5)	C18—N4	1.423 (6)
C1—O1	1.274 (5)	C19—C20	1.386 (6)
C1—C2	1.503 (6)	C19—H19	0.9300
C2—C7	1.383 (6)	C20—C21	1.385 (6)
C2—C3	1.393 (6)	C20—C22	1.499 (6)
C3—C4	1.384 (6)	C21—H21	0.9300
C3—H3	0.9300	C22—O9	1.256 (5)
C4—C5	1.394 (6)	C22—O8	1.265 (5)
C4—C8	1.498 (6)	C23—O10	1.224 (6)
C5—C6	1.364 (6)	C23—N4	1.341 (6)
C5—H5	0.9300	C23—C24	1.485 (7)
C6—C7	1.382 (6)	C24—C28	1.376 (8)
C6—N2	1.416 (6)	C24—C25	1.382 (8)
C7—H7	0.9300	C25—C26	1.365 (7)
C8—O3	1.255 (6)	C25—H25	0.9300
C8—O4	1.260 (5)	C26—N3	1.343 (8)
C9—O5	1.225 (6)	C26—H26	0.9300
C9—N2	1.329 (6)	C27—N3	1.335 (7)
C9—C10	1.503 (7)	C27—C28	1.388 (7)
C10—C14	1.374 (7)	C27—H27	0.9300
C10—C11	1.378 (7)	C28—H28	0.9300
C11—C12	1.371 (7)	N2—H2	0.8600
C11—H11	0.9300	N4—H4	0.8600
C12—N1	1.324 (7)	O1W—H1WB	0.8500
C12—H12	0.9300	O1W—H1WA	0.8500
			
N1^i^—Ag1—N3	121.79 (16)	O5—C9—C10	121.0 (4)
N1^i^—Ag1—O7^ii^	124.05 (14)	N2—C9—C10	115.1 (4)
N3—Ag1—O7^ii^	106.74 (14)	C14—C10—C11	117.8 (5)
O9^iii^—Ce1—O8^iv^	133.09 (10)	C14—C10—C9	122.9 (4)
O9^iii^—Ce1—O6	142.14 (11)	C11—C10—C9	119.1 (5)
O8^iv^—Ce1—O6	76.29 (11)	C12—C11—C10	118.6 (5)
O9^iii^—Ce1—O3^v^	83.67 (11)	C12—C11—H11	120.7
O8^iv^—Ce1—O3^v^	141.72 (10)	C10—C11—H11	120.7
O6—Ce1—O3^v^	77.23 (11)	N1—C12—C11	123.9 (5)
O9^iii^—Ce1—O2^vi^	77.92 (11)	N1—C12—H12	118.0
O8^iv^—Ce1—O2^vi^	105.14 (11)	C11—C12—H12	118.0
O6—Ce1—O2^vi^	122.35 (10)	N1—C13—C14	123.5 (5)
O3^v^—Ce1—O2^vi^	67.59 (10)	N1—C13—H13	118.2
O9^iii^—Ce1—O1	69.33 (10)	C14—C13—H13	118.2
O8^iv^—Ce1—O1	69.60 (10)	C10—C14—C13	119.1 (5)
O6—Ce1—O1	113.51 (10)	C10—C14—H14	120.4
O3^v^—Ce1—O1	147.60 (10)	C13—C14—H14	120.4
O2^vi^—Ce1—O1	120.84 (10)	O6—C15—O7	122.8 (4)
O9^iii^—Ce1—O1W	75.72 (10)	O6—C15—C16	116.8 (4)
O8^iv^—Ce1—O1W	119.75 (11)	O7—C15—C16	120.4 (4)
O6—Ce1—O1W	67.66 (10)	C21—C16—C17	119.9 (4)
O3^v^—Ce1—O1W	73.47 (10)	C21—C16—C15	120.7 (4)
O2^vi^—Ce1—O1W	134.75 (11)	C17—C16—C15	119.4 (4)
O1—Ce1—O1W	82.50 (10)	C18—C17—C16	120.4 (4)
O9^iii^—Ce1—O1^vi^	79.04 (10)	C18—C17—H17	119.8
O8^iv^—Ce1—O1^vi^	69.80 (10)	C16—C17—H17	119.8
O6—Ce1—O1^vi^	138.81 (10)	C17—C18—C19	119.6 (4)
O3^v^—Ce1—O1^vi^	117.28 (10)	C17—C18—N4	119.0 (4)
O2^vi^—Ce1—O1^vi^	49.96 (10)	C19—C18—N4	121.3 (4)
O1—Ce1—O1^vi^	75.80 (10)	C20—C19—C18	120.4 (4)
O1W—Ce1—O1^vi^	151.22 (9)	C20—C19—H19	119.8
O9^iii^—Ce1—O7	147.39 (10)	C18—C19—H19	119.8
O8^iv^—Ce1—O7	72.15 (10)	C21—C20—C19	119.7 (4)
O6—Ce1—O7	50.26 (9)	C21—C20—C22	120.2 (4)
O3^v^—Ce1—O7	69.70 (10)	C19—C20—C22	120.0 (4)
O2^vi^—Ce1—O7	74.76 (10)	C16—C21—C20	120.0 (4)
O1—Ce1—O7	141.32 (10)	C16—C21—H21	120.0
O1W—Ce1—O7	112.36 (9)	C20—C21—H21	120.0
O1^vi^—Ce1—O7	96.35 (9)	O9—C22—O8	125.2 (4)
O9^iii^—Ce1—C1^vi^	77.77 (11)	O9—C22—C20	117.9 (4)
O8^iv^—Ce1—C1^vi^	87.39 (12)	O8—C22—C20	116.9 (4)
O6—Ce1—C1^vi^	134.58 (11)	O10—C23—N4	121.8 (5)
O3^v^—Ce1—C1^vi^	91.94 (12)	O10—C23—C24	119.4 (5)
O2^vi^—Ce1—C1^vi^	24.43 (11)	N4—C23—C24	118.8 (5)
O1—Ce1—C1^vi^	99.20 (11)	C28—C24—C25	117.2 (5)
O1W—Ce1—C1^vi^	150.89 (12)	C28—C24—C23	124.7 (5)
O1^vi^—Ce1—C1^vi^	25.54 (11)	C25—C24—C23	118.1 (5)
O7—Ce1—C1^vi^	84.49 (10)	C26—C25—C24	120.1 (6)
O9^iii^—Ce1—Ce1^vi^	70.04 (7)	C26—C25—H25	119.9
O8^iv^—Ce1—Ce1^vi^	63.92 (7)	C24—C25—H25	119.9
O6—Ce1—Ce1^vi^	137.33 (8)	N3—C26—C25	122.7 (6)
O3^v^—Ce1—Ce1^vi^	144.99 (8)	N3—C26—H26	118.6
O2^vi^—Ce1—Ce1^vi^	84.17 (7)	C25—C26—H26	118.6
O1—Ce1—Ce1^vi^	39.05 (7)	N3—C27—C28	121.9 (6)
O1W—Ce1—Ce1^vi^	119.27 (7)	N3—C27—H27	119.1
O1^vi^—Ce1—Ce1^vi^	36.76 (6)	C28—C27—H27	119.1
O7—Ce1—Ce1^vi^	123.63 (7)	C24—C28—C27	120.2 (5)
C1^vi^—Ce1—Ce1^vi^	60.81 (9)	C24—C28—H28	119.9
O2—C1—O1	122.0 (4)	C27—C28—H28	119.9
O2—C1—C2	119.2 (4)	C13—N1—C12	116.9 (5)
O1—C1—C2	118.8 (4)	C13—N1—Ag1^i^	123.5 (4)
O2—C1—Ce1^vi^	56.7 (2)	C12—N1—Ag1^i^	116.1 (3)
O1—C1—Ce1^vi^	65.3 (2)	C9—N2—C6	125.4 (4)
C2—C1—Ce1^vi^	175.4 (3)	C9—N2—H2	117.3
C7—C2—C3	120.4 (4)	C6—N2—H2	117.3
C7—C2—C1	119.1 (4)	C27—N3—C26	117.9 (5)
C3—C2—C1	120.4 (4)	C27—N3—Ag1	121.3 (4)
C4—C3—C2	119.2 (4)	C26—N3—Ag1	120.8 (4)
C4—C3—H3	120.4	C23—N4—C18	122.0 (4)
C2—C3—H3	120.4	C23—N4—H4	119.0
C3—C4—C5	119.6 (4)	C18—N4—H4	119.0
C3—C4—C8	120.7 (4)	C1—O1—Ce1	152.6 (3)
C5—C4—C8	119.5 (4)	C1—O1—Ce1^vi^	89.1 (3)
C6—C5—C4	120.9 (4)	Ce1—O1—Ce1^vi^	104.20 (10)
C6—C5—H5	119.6	C1—O2—Ce1^vi^	98.9 (3)
C4—C5—H5	119.6	C8—O3—Ce1^vii^	137.7 (3)
C5—C6—C7	120.0 (4)	C15—O6—Ce1	97.7 (3)
C5—C6—N2	122.7 (4)	C15—O7—Ag1^viii^	119.3 (3)
C7—C6—N2	117.2 (4)	C15—O7—Ce1	87.7 (2)
C6—C7—C2	119.9 (4)	Ag1^viii^—O7—Ce1	107.58 (12)
C6—C7—H7	120.1	C22—O8—Ce1^iv^	131.5 (3)
C2—C7—H7	120.1	C22—O9—Ce1^ix^	129.5 (3)
O3—C8—O4	124.5 (4)	Ce1—O1W—H1WB	108.0
O3—C8—C4	118.0 (4)	Ce1—O1W—H1WA	107.2
O4—C8—C4	117.4 (4)	H1WB—O1W—H1WA	107.1
O5—C9—N2	123.9 (4)		
			
O2—C1—C2—C7	−160.8 (4)	N1^i^—Ag1—N3—C26	140.5 (4)
O1—C1—C2—C7	19.1 (6)	O7^ii^—Ag1—N3—C26	−10.4 (5)
O2—C1—C2—C3	17.6 (7)	O10—C23—N4—C18	7.5 (8)
O1—C1—C2—C3	−162.4 (4)	C24—C23—N4—C18	−172.8 (4)
C7—C2—C3—C4	−0.3 (7)	C17—C18—N4—C23	124.9 (5)
C1—C2—C3—C4	−178.7 (4)	C19—C18—N4—C23	−52.0 (7)
C2—C3—C4—C5	−0.5 (7)	O2—C1—O1—Ce1	−118.1 (6)
C2—C3—C4—C8	173.2 (4)	C2—C1—O1—Ce1	62.0 (8)
C3—C4—C5—C6	0.5 (7)	Ce1^vi^—C1—O1—Ce1	−120.2 (6)
C8—C4—C5—C6	−173.3 (4)	O2—C1—O1—Ce1^vi^	2.1 (4)
C4—C5—C6—C7	0.3 (7)	C2—C1—O1—Ce1^vi^	−177.8 (4)
C4—C5—C6—N2	177.7 (4)	O9^iii^—Ce1—O1—C1	−159.6 (6)
C5—C6—C7—C2	−1.0 (7)	O8^iv^—Ce1—O1—C1	43.6 (6)
N2—C6—C7—C2	−178.6 (4)	O6—Ce1—O1—C1	−20.4 (6)
C3—C2—C7—C6	1.0 (7)	O3^v^—Ce1—O1—C1	−124.1 (6)
C1—C2—C7—C6	179.4 (4)	O2^vi^—Ce1—O1—C1	139.5 (6)
C3—C4—C8—O3	29.7 (7)	O1W—Ce1—O1—C1	−82.1 (6)
C5—C4—C8—O3	−156.5 (4)	O1^vi^—Ce1—O1—C1	117.0 (7)
C3—C4—C8—O4	−147.4 (4)	O7—Ce1—O1—C1	34.7 (7)
C5—C4—C8—O4	26.3 (6)	C1^vi^—Ce1—O1—C1	127.3 (6)
O5—C9—C10—C14	139.0 (5)	Ce1^vi^—Ce1—O1—C1	117.0 (7)
N2—C9—C10—C14	−38.8 (7)	O9^iii^—Ce1—O1—Ce1^vi^	83.44 (12)
O5—C9—C10—C11	−36.7 (7)	O8^iv^—Ce1—O1—Ce1^vi^	−73.38 (11)
N2—C9—C10—C11	145.5 (5)	O6—Ce1—O1—Ce1^vi^	−137.43 (11)
C14—C10—C11—C12	−1.0 (8)	O3^v^—Ce1—O1—Ce1^vi^	118.93 (17)
C9—C10—C11—C12	174.9 (5)	O2^vi^—Ce1—O1—Ce1^vi^	22.50 (16)
C10—C11—C12—N1	−2.4 (10)	O1W—Ce1—O1—Ce1^vi^	160.95 (12)
C11—C10—C14—C13	2.4 (8)	O1^vi^—Ce1—O1—Ce1^vi^	0.0
C9—C10—C14—C13	−173.4 (5)	O7—Ce1—O1—Ce1^vi^	−82.34 (16)
N1—C13—C14—C10	−0.5 (10)	C1^vi^—Ce1—O1—Ce1^vi^	10.33 (13)
O6—C15—C16—C21	151.8 (5)	O1—C1—O2—Ce1^vi^	−2.3 (5)
O7—C15—C16—C21	−28.0 (7)	C2—C1—O2—Ce1^vi^	177.6 (3)
O6—C15—C16—C17	−26.0 (7)	O4—C8—O3—Ce1^vii^	26.3 (8)
O7—C15—C16—C17	154.2 (5)	C4—C8—O3—Ce1^vii^	−150.6 (3)
C21—C16—C17—C18	2.2 (7)	O7—C15—O6—Ce1	−13.1 (5)
C15—C16—C17—C18	180.0 (4)	C16—C15—O6—Ce1	167.0 (4)
C16—C17—C18—C19	−1.7 (8)	O9^iii^—Ce1—O6—C15	−129.0 (3)
C16—C17—C18—N4	−178.7 (4)	O8^iv^—Ce1—O6—C15	84.7 (3)
C17—C18—C19—C20	0.0 (7)	O3^v^—Ce1—O6—C15	−67.4 (3)
N4—C18—C19—C20	176.9 (5)	O2^vi^—Ce1—O6—C15	−14.7 (3)
C18—C19—C20—C21	1.2 (7)	O1—Ce1—O6—C15	144.9 (3)
C18—C19—C20—C22	−175.0 (4)	O1W—Ce1—O6—C15	−144.5 (3)
C17—C16—C21—C20	−1.0 (7)	O1^vi^—Ce1—O6—C15	49.7 (4)
C15—C16—C21—C20	−178.7 (4)	O7—Ce1—O6—C15	6.7 (3)
C19—C20—C21—C16	−0.7 (7)	C1^vi^—Ce1—O6—C15	12.6 (4)
C22—C20—C21—C16	175.4 (4)	Ce1^vi^—Ce1—O6—C15	105.9 (3)
C21—C20—C22—O9	165.3 (4)	O6—C15—O7—Ag1^viii^	121.0 (4)
C19—C20—C22—O9	−18.5 (6)	C16—C15—O7—Ag1^viii^	−59.2 (5)
C21—C20—C22—O8	−15.4 (7)	O6—C15—O7—Ce1	12.0 (5)
C19—C20—C22—O8	160.8 (4)	C16—C15—O7—Ce1	−168.1 (4)
O10—C23—C24—C28	137.3 (6)	O9^iii^—Ce1—O7—C15	120.7 (3)
N4—C23—C24—C28	−42.4 (8)	O8^iv^—Ce1—O7—C15	−93.3 (3)
O10—C23—C24—C25	−40.9 (8)	O6—Ce1—O7—C15	−6.6 (3)
N4—C23—C24—C25	139.4 (5)	O3^v^—Ce1—O7—C15	83.5 (3)
C28—C24—C25—C26	−0.8 (8)	O2^vi^—Ce1—O7—C15	154.8 (3)
C23—C24—C25—C26	177.5 (5)	O1—Ce1—O7—C15	−84.5 (3)
C24—C25—C26—N3	−0.3 (9)	O1W—Ce1—O7—C15	22.2 (3)
C25—C24—C28—C27	1.4 (8)	O1^vi^—Ce1—O7—C15	−159.7 (3)
C23—C24—C28—C27	−176.8 (5)	C1^vi^—Ce1—O7—C15	177.6 (3)
N3—C27—C28—C24	−0.9 (9)	Ce1^vi^—Ce1—O7—C15	−133.1 (3)
C14—C13—N1—C12	−2.7 (10)	O9^iii^—Ce1—O7—Ag1^viii^	0.7 (2)
C14—C13—N1—Ag1^i^	154.9 (5)	O8^iv^—Ce1—O7—Ag1^viii^	146.62 (14)
C11—C12—N1—C13	4.2 (10)	O6—Ce1—O7—Ag1^viii^	−126.60 (18)
C11—C12—N1—Ag1^i^	−155.1 (5)	O3^v^—Ce1—O7—Ag1^viii^	−36.53 (12)
O5—C9—N2—C6	−7.6 (7)	O2^vi^—Ce1—O7—Ag1^viii^	34.77 (12)
C10—C9—N2—C6	170.2 (4)	O1—Ce1—O7—Ag1^viii^	155.43 (12)
C5—C6—N2—C9	45.2 (7)	O1W—Ce1—O7—Ag1^viii^	−97.83 (13)
C7—C6—N2—C9	−137.3 (5)	O1^vi^—Ce1—O7—Ag1^viii^	80.25 (12)
C28—C27—N3—C26	−0.2 (8)	C1^vi^—Ce1—O7—Ag1^viii^	57.59 (13)
C28—C27—N3—Ag1	−179.0 (4)	Ce1^vi^—Ce1—O7—Ag1^viii^	106.86 (10)
C25—C26—N3—C27	0.8 (9)	O9—C22—O8—Ce1^iv^	65.6 (6)
C25—C26—N3—Ag1	179.6 (4)	C20—C22—O8—Ce1^iv^	−113.6 (4)
N1^i^—Ag1—N3—C27	−40.7 (5)	O8—C22—O9—Ce1^ix^	−41.8 (6)
O7^ii^—Ag1—N3—C27	168.3 (4)	C20—C22—O9—Ce1^ix^	137.4 (3)

Symmetry codes: (i) −*x*+2, −*y*+1, −*z*+3; (ii) *x*, *y*, *z*+1; (iii) *x*, *y*+1, *z*; (iv) −*x*+1, −*y*, −*z*+1; (v) *x*+1, *y*, *z*; (vi) −*x*+1, −*y*+1, −*z*+1; (vii) *x*−1, *y*, *z*; (viii) *x*, *y*, *z*−1; (ix) *x*, *y*−1, *z*.

### Hydrogen-bond geometry (Å, °)

**Table:**

*D*—H···*A*	*D*—H	H···*A*	*D*···*A*	*D*—H···*A*
O1W—H1WA···O5^x^	0.85	2.08	2.836 (5)	147
O1W—H1WB···O4^v^	0.85	2.01	2.691 (6)	137
N2—H2···O10^iii^	0.86	2.01	2.788 (7)	149
N4—H4···O4^x^	0.86	2.08	2.926 (6)	167

Symmetry codes: (x) −*x*+1, −*y*+1, −*z*+2; (v) *x*+1, *y*, *z*; (iii) *x*, *y*+1, *z*.
